# The Relationship Between Problematic Use of Social Networks, Perceived Stress, Distraction, and Self-Management in Nursing Students: A Cross-Sectional Study

**DOI:** 10.3390/nursrep16030086

**Published:** 2026-03-02

**Authors:** Gema López-Gutiérrez, Vanesa Gutiérrez-Puertas, Blanca Gómez-Guerrero, Lorena Gutiérrez-Puertas

**Affiliations:** 1 Department of Neurology, Torrecardenas University Hospital, Calle Hermandad de Donantes de Sangre, s/n, 04009 Almeria, AN, Spain; gema.lopez.gutierrez.sspa@juntadeandalucia.es; 2 Department of Hematology, Torrecardenas University Hospital, Calle Hermandad de Donantes de Sangre, s/n, 04009 Almeria, AN, Spain; blanca.gomez.guerrero.sspa@juntadeandalucia.es; 3 Department of Nursing, Physiotherapy and Medicine, Faculty of Health Science, University of Almeria, Ctra. Sacramento s/n, La Cañada de San Urbano, 04120 Almeria, AN, Spain; lgp524@ual.es; 4 Research Group PAIDI-TIC 019 “Electronic Communications and Telemedicine”, University of Almeria, Ctra. Sacramento s/n, La Cañada de San Urbano, 04120 Almeria, AN, Spain; 5 Department of Nephrology, Poniente University Hospital, Ctra. Almerimar, s/n, El Ejido, 04700 Almeria, AN, Spain; 6 Research Group PAIDI-HUM 061 “Experimental and Applied Neuropsychology”, University of Almeria, Ctra. Sacramento s/n, La Cañada de San Urbano, 04120 Almeria, AN, Spain

**Keywords:** attention, nursing students, self-management, smartphone, social networks, stress

## Abstract

**Background/Objective**: Nursing students commonly use social networking sites during clinical practicums, and excessive use may interfere with their attention, performance, and training during clinical placements. However, the relationship between problematic social networking use, perceived stress, distraction, and self-management of activities during clinical practice among nursing students has not been explored in depth. The aim of this study was to analyze the relationship between problematic use of social networking sites, perceived stress, smartphone-related distraction, and self-management among nursing students during clinical practicums. **Methods**: A cross-sectional design was carried out. The sample consisted of 340 nursing students. Data were collected between September and November 2025 using a sociodemographic form, the WhatsApp Negative Impact Scale, Perceived Stress Scale, Distraction Smartphone Use during Clinical Practice Scale, and Self-Control and Self-Management Scale. The data were analyzed using SPSS 28. Regression analysis was performed to define the factor of the problematic use of social networks and the relationship between the scales. The STROBE Checklist was used when preparing the manuscript. **Results:** A positive relationship was found between problematic social networks use and perceived stress (*p* < 0.001) and distraction (*p* < 0.001), and there was a negative relationship with self-management (*p* < 0.001). A negative relationship was found between perceived stress and self-management (*p* < 0.001). After regression analysis, more problematic social network use consisted of those who spent more time daily using the smartphone (*p* < 0.001), had more distraction associated with smartphone use during clinical practicum (*p* < 0.001), had more perceived stress (*p* < 0.001), those whose last place performing the practicum was the hospital (*p* = 0.006), and those whose sex was male (*p* = 0.026). **Conclusions:** The results obtained from this study indicated an association between problematic Social networksuse and increased perceived stress and distraction, as well as decreased self-management activities during clinical practicum. In line with these findings, perceived stress was negatively associated with self-management; nursing students reporting lower perceived stress also reported a greater capacity for self-management of clinical activities.

## 1. Introduction

Social networks (SNSs) provide quick and easy access to information and numerous entertainment or socializing activities via the Internet, which has increased their use [1]. Worldwide, it is estimated that SNS users spend more than two hours a day using SNSs [2]. Spain is one of the largest SNS markets in Western Europe with more than 40 million SNS users [3]. Specifically, WhatsApp is used by 91% of Spanish Internet users, making it the most used SNS, with smartphones being the main means of access to this SNS. WhatsApp allows users to send and receive text messages, voice messages, images, videos, and documents and allow users to create groups [2]. Systematic reviews consider WhatsApp to be an SNS because it allows for online interaction and the creation of communities and group communication, as well as the sharing of information and images that can be commented on [4]. Studies on the problematic use of social media platforms analyze WhatsApp alongside other SNS platforms (such as Facebook, Instagram, or TikTok), which supports its inclusion as an SNS [5].

SNSs in the clinical setting can positively impact professional development when it is used to exchange and look up information or to publish advances in the health field [6]. Furthermore, SNSs can be used as educational resources to improve the training process of nursing students in clinical settings [7]. However, when nursing students use SNSs for personal matters, it can negatively impact their clinical and learning environment [8] and patient confidentiality or safety [9]. Several studies have explored the problematic use of new technologies by nursing students [10,11,12]. Particularly, it has been determined that the level of addiction to the Internet [9] and smartphones are high [13], indicating that using SNSs is the activity that nursing students do most with their smartphones [10,11,12]. Problematic SNS use refers to excessive or uncontrolled use of these, which can cause anxiety when unable to use them, fear of being unavailable, dependence on them to relieve negative emotions, and interference with interpersonal relationships or work activities, leading to symptoms similar to those of an addiction [14]. In addition, nursing students indicate using their smartphone for personal matters in clinical settings [8]. Approximately 22% of nursing students have problematic smartphone use, with the main activity being the use of SNSs [13]. SNS addiction significantly predicts lower academic performance and increased procrastination in nursing students, negatively affecting their attention, motivation, and academic performance [15,16,17]. Distraction caused by mobile phones in the clinical setting is defined as the interruption of a clinical activity due to internal or external causes related to the individual’s use of a mobile phone [18]. Consequently, this source of distraction [12] may interrupt their principal duties such as providing health care and therefore interfere with self-management of clinical activities [11]. Among nurses, increased mobile phone use during shift work is associated with increased interruptions and distractions during clinical work, as well as a decrease in time spent on clinical tasks, negatively affecting efficiency, privacy, and patient safety [19,20]. Among nurses, problematic mobile phone use is associated with a decrease in effective communication with patients, attention span, perceived clinical decision-making ability [21,22,23], increased procrastination, accumulation of relevant clinical tasks, and reduced patient care time [24]. However, there are no prior studies on nursing students that study this association.

Self-management is defined as the ability to have a high level of control over oneself while working, constituting an essential characteristic for successful work performance [15]. Likewise, self-management allows the individual to focus their attention on the task being carried out and effectively complete it without being distracted by other activities such as using their smartphones to access SNSs [25]. Several studies have examined the relationship between SNS addiction and professional performance in nurses. Specifically, Javed et al. [26] demonstrated that SNS addiction negatively affects nurses’ performance, mediated by task distraction, while self-management moderates this relationship. Furthermore, Hoşgör et al. [19] found a significant negative correlation between social media addiction and work engagement among nurses. Additionally, excessive SNS use by nursing professionals has been shown to increase fatigue [27] and perceived stress [11]. The stress perceived among nursing students during clinical practice refers to factors such as lack of clinical skills; heavy workload; relationship problems with peers, instructors, and nursing staff; time pressure to perform nursing tasks; and work pressure [28]. Chronic or excessive perceived stress decreases the clinical performance of nursing students, which can ultimately compromise the quality of patient care [29]. Previous studies show that nursing students display high levels of perceived stress during their clinical practicums [30,31]. In particular, the perceived stress of nursing students may increase due to uncertainty, lack of capacity to perform clinical procedures, workload, clinical evaluations, and relationships with other nursing professionals or patients [32]. Furthermore, nursing students perceived stress may negatively affect their academic and clinical performance [11,33]. Perceived stress generated by university life could lead some to become addicted to the Internet [34], therefore being a potential predictor of Internet abuse, though this association has yet to be explored.

According to the above, while related evidence exists on problematic SNS use, perceived stress, and smartphone distraction among nursing students [12,35,36], no prior study has integrated all these variables with self-management during clinical practicums. Therefore, the aim of this study was to analyze the relationship between problematic use of social networking sites, perceived stress, smartphone-related distraction, and self-management among nursing students during clinical practicums. Specifically, we aimed to: (1) describe participants’ sociodemographic and practicum-related characteristics and the main study variables; (2) analyze the association between problematic SNS use, perceived stress, smartphone-related distraction, and self-management; and (3) identify factors associated with problematic SNS use during clinical practicums.

## 2. Materials and Methods

### 2.1. Study Design, Participants, and Setting

A cross-sectional study was conducted. The Reporting of Observational Studies in Epidemiology (STROBE) guidelines were followed to ensure the quality of the study [37]. The study was performed on students enrolled in a nursing degree at the University of Almeria (Spain) in the 2024–2025 academic year. The sample was selected through convenience sampling. A nursing degree in Almeria (Spain) consists of four academic years. The practicum subjects were in their second, third, and fourth academic years. The study population involved second, third, and fourth year nursing student who had completed at least one practicum subject. G*Powert software version 3.1.9.7 (Heinrich-Heine-Universität Düsseldorf, Düsseldorf, Germany) [38] was used to calculate the sample size and estimate the sample required, considering a minimum correlation of 0.3 between scores on the questionnaires, a significance level (α) of 0.05, and a power (1 − β) of 0.80; the minimum number of participants required for this study was 67. Finally, 340 nursing students participated, of whom 106 corresponded to the second year, 103 to the third year, and 131 to the fourth year of Nursing degree. The inclusion criteria established were: (i) be enrolled in a practicum subject; (ii) have completed at least one practicum subject; (iii) use the WhatsApp SNS in the clinical setting. The exclusion criterion was being an exchange student. Data collection took place between September and November 2025.

### 2.2. Measurements

The data of the study were collected using four validated instruments: WhatsApp Negative Impact Scale (WANIS) [39], Perceived Stress Scale (PSS) [40], Distraction Smartphone Use during Clinical Practice Scale [10], and Self-Control and Self-Management Scale (SCMS) [15]. Permission was requested from the authors of the instruments who expressed agreement to use them. A questionnaire of sociodemographic variables was developed ad hoc.

#### 2.2.1. Sociodemographic Form

The sociodemographic data collected were sex (male or female), age, academic year (2nd, 3rd or 4th year), the last place where their clinical practicums (hospital or health clinic) were carried out, and the amount of time of daily use of the smartphone (<1, 1–3, 3–5, >5 h/day).

#### 2.2.2. The WhatsApp Negative Impact Scale (WANIS)

WANIS was used to evaluate problematic use of the WhatsApp SNS [39]. The instrument contains 37 items distributed in three factors: Negative Consequences of WhatsApp Use (15 items), Controlling relationships through WhatsApp (10 items), and Problematic use of WhatsApp (12 items). Each item is scored on a Likert-type scale from 0 “completely disagree” to 3 “completely agree” (e.g., “When I’m with my friends, I pay more attention to WhatsApp than to them.”). The WANIS score ranges from 0 to 111. Higher scores indicate greater problematic use of the WhatsApp SNS. WANIS has a Cronbach’s alpha of 0.95; the Cronbach’s alpha for each scale dimension was 0.93 for factor I (Negative Consequences of WhatsApp Use), 0.85 for factor II (Controlling Intimate Relationships through WhatsApp), and 0.87 for factor III (Problematic Use of WhatsApp) [39]. In this study, the Cronbach’s alpha was 0.91.

#### 2.2.3. Perceived Stress Scale (PSS)

The PSS was developed by Cohen et al. [40]; this study employed the European Spanish version validated by Remor et al. [41]. The PSS evaluates the overall perception of stress [40]. The scale is composed of 14 items (7 positively worded items and 7 negatively worded items). The items are assessed on a Likert-type scale from 0 to 4 “never to very often”, respectively, (e.g., “In the last month, how often have you been upset because of something that happened unexpectedly?”). The total score from the scale ranges from 0 to 56. High scores indicate greater levels of perceived stress. The Cronbach’s alpha of the questionnaire was 0.84 [41]. In this study, it had a Cronbach’s alpha of 0.81.

#### 2.2.4. Distraction Smartphone Use During Clinical Practice Scale (DSCS)

Distraction Smartphone Use during Clinical Practice Scale assesses the distraction caused by smartphone use in a clinical setting [10], consisting of five items valued on a Likert-type scale, ranging from 0 “never” to 4 “always” (e.g., “Have you been distracted by own use of a smartphone during clinical practicum?”). The total score ranges from 0 to 20. Higher scores correspond to greater levels of distraction. The Cronbach’s alpha reported by the original authors was 0.80 [8]. In this study, the scale was translated and culturally adapted following the guidelines by Beaton et al. [42] (forward translation, synthesis, back-translation, expert committee review, and pilot testing in sample of nursing students). A Cronbach’s alpha of 0.81 was calculated in this study.

#### 2.2.5. Self-Control and Self-Management Scale (SCMS)

The SCMS was used to assess self-management [15]. This scale consists of 16 items (11 positively worded items and 5 negatively worded items) that were rated using a Likert-type scale ranging from 0 “very undescriptive of me” to 5 “very descriptive of me” (e.g., “When I work toward something, it gets all my attention.”) and three factors: Self-Monitoring (Items 1 to 6), Self-Evaluation (Items 7 to 11), and Self-Reinforcing (Items 12 to 16). The total score from the scale ranges from 0 to 80. Higher scores indicate a greater level of self-management. The Cronbach’s alpha of this scale was 0.81; the Cronbach’s alpha for each scale dimension was 0.74 for the self-monitoring subscale, 0.75 for the self-evaluation subscale and 0.78 for the self-reinforcing subscale [12]. In this study, the scale was translated and culturally adapted following the guidelines by Beaton et al. [42] (forward translation, synthesis, back-translation, expert committee review, and pilot testing in sample of nursing students). The Cronbach’s alpha was 0.85 for this study.

### 2.3. Ethical Considerations and Data Collection

The study was approved by the University Institutional Review Board (EFM 442.25, July 2025). No incentives or payments were offered to nursing students for their participation. Before the beginning of the study, the participants had to sign an informed consent form. Participants were previously informed of the objective of the study, the voluntary nature, and the possibility of leaving the study at any time, as well as the anonymous, confidential treatment of their data. The guidelines established in the Helsinki declaration were followed at all times.

Once ethical approval was obtained, nursing students enrolled in the second, third, and fourth year of the nursing degree (The nursing degree in Almeria consists of four academic years) were invited to participate in the study by email. In an in-person meeting, the purpose of the study was explicitly explained to all prospective participants, as well as its voluntary nature. The students who wanted to participate were given an informed consent form to sign in order to participate in the study. A survey instrument, in the form of paper-based questionnaires, were distributed by the researcher to those who had consented to participate. The data collection process was conducted in a classroom of the Faculty of Health Sciences during the student’s free time to ensure minimal disruption to their regular academic activities. Participants completed the questionnaire over a period of approximately 15–20 min. Once all questionnaires had been completed, participants deposited them in a box located in the room to ensure anonymity. The questionnaires were then collected by the researcher responsible for data management. The questionnaires were stored in a locked closet accessible only to the researchers who participated in the collection and analysis of the data. This was conducted to assure students of the confidentiality of their responses and their use solely for the purposes of this research study. At the end of the session, the researcher thanked the participants for their collaboration. The data collection took place between September and November 2025.

### 2.4. Data Analysis

The statistical program IBM SPSS^®^ Statistics version 28 (IBM Inc., Armonk, NY, USA) was used for data analysis. A descriptive analysis of the sociodemographic characteristics was carried out, calculating frequencies and percentages for categorical variables; mean and standard deviation were calculated to describe quantitative variables. After testing the sample distribution through a Kolmogorov–Smirnov test, Q-Q Plots, and histogram analyses, a non-parametric test was used. It was observed that the variables did not follow a normal distribution. Consequently, Spearman’s correlation was used to analyze the association between problematic SNS use, PSS, DSCS, and SCMS of clinical activities.

Factors that influenced problematic use of SNSs were analyzed through multiple regression. Before estimating the model, compliance with the assumptions of multiple linear regression was verified using graphical procedures and diagnostic statistics, including the adequacy of the linear relationship between variables, the normality of residuals, homoscedasticity, independence of errors, and the absence of multicollinearity. *p*< 0.05 was considered significant.

## 3. Results

### 3.1. Sociodemographic Characteristics of the Sample

Of the 340 nursing students, 15.60 (*n* = 53) were male and 84.40 (*n* = 287) were female. The mean age was 23.00 (*SD* = 4.58). The rest of the sociodemographic characteristics of the sample can be seen in Table 1.

### 3.2. Correlations of WhatsApp Negative Impact Scale, Perceived Stress Scale, Distraction Smartphone Use During Clinical Practice, and Self-Control and Self-Management Scale

The mean score for the WhatsApp Negative Impact Scale was 53.84 ± 16.14 (ranging from 0 to 111). The average Perceived Stress Scale score was 34.54 ± 6.60 (ranging from 0 to 56). Regarding Distraction Smartphone Use during Clinical Practice was 7.74 ± 3.14 (ranging from 0 to 20). The mean score for the Self-Control and Self-Management Scale was 55.63 ± 8.00 (ranging from 0 to 80).

The results of the association between the WhatsApp Negative Impact Scale, Perceived Stress Scale, Distraction Smartphone Use during Clinical Practice, and Self-Management Scale are shown in Table 2. Specifically, a positive association was found between the WhatsApp Negative Impact Scale and Perceived Stress Scale (*r_s_* = 0.26, *p* < 0.001); a positive association was found between the WhatsApp Negative Impact Scale and distraction (*r_s_* = 0.25, *p* < 0.001); a negative association was found between the WhatsApp Negative Impact Scale and Self-Control and Self-Management Scale (*r_s_* = −0.19, *p* < 0.001); and a negative association was found between the Perceived Stress Scale and Self-Control and Self-Management Scale (*r_s_* = −0.28, *p* < 0.001).

### 3.3. Regression Correlations with Demographic Data

The multiple regression model was statistically significant and shows a proportion of variance (R^2^ = 0.19, F (5, 334) = 4.97, *p* = 0.026; adjusted R^2^ = 0.18). In the multiple regression analysis, daily time spent on smartphone, distraction, perceived stress, and last place of practicum was carried out and sex explained 19.1% of problematic SNS use (Table 3).

Nursing students with more problematic SNS use were the ones who spent more time daily using the smartphone (*β* = 0.21, *p* < 0.001), had more distraction associated with smartphone use during clinical practicum (*β* = 0.22, *p* < 0.001), had more perceived stress (*β* = 0.19, *p* < 0.001), last place where they performed the practicum being the hospital (*β* = −0.146, *p* = 0.006), and whose sex was male (*β* = −0.124, *p* = 0.026).

## 4. Discussion

The aim of this study was to analyze the relationship between problematic use of SNSs, perceived stress, smartphone-related distraction, and self-management among nursing students during clinical practicums. Previous studies have explored the relationship between several variables and problematic use of SNSs in nursing students; to the best of our knowledge, the interrelationship between the variables in the present study has not been investigated. In line with our objective, we described levels of the negative impact of SNS use, perceived stress, smartphone-related distraction during clinical practice, and self-management; examined their associations; and identified factors associated with the negative impact of SNS use. The negative impact of SNS use was associated with higher perceived stress and greater smartphone-related distraction and with lower self-management. However, smartphone-related distraction showed no association with perceived stress or self-control/self-management.

The scores for problematic SNS use suggested a moderate negative impact, and most students reported more than three hours of mobile phone use per day. Previous studies of nursing students report frequent smartphone use during clinical practice, with a minority reporting feeling distracted despite widespread observation of others’ use [10,12,35]. Furthermore, several studies have indicated that the activity most frequently performed by nursing students with their smartphones is accessing social media [11,13]. Similarly, problematic smartphone use has been linked to excessive social media use [43] and has been associated with poorer mental health indicators, including stress-related symptoms [44,45]. Perceived stress was positively associated with problematic SNS use, suggesting that higher stress may coincide with more problematic SNS use during clinical practice. However, previous studies show higher levels of perceived stress among nursing students [30,45]. This could be due to the fact that the students included in this study were enrolled in the final years of the nursing degree. In particular, the stress perceived by nursing students decreases as they begin to engage in clinical situations that provide opportunities to improve their clinical skills [31]. Regarding distraction associated with smartphone use, scores were low on average, but distraction associated with mobile phone use during clinical practice increased with problematic SNS use and longer daily mobile phone use. This pattern is consistent with evidence that nursing students report low levels of perceived self-distraction, while reporting that they regularly witness other students and nursing professionals being distracted by their smartphones in the clinical setting [10,12,35]. This could be due to the fact that nursing students use their smartphones in clinical settings to consult information, calculate medication doses, access university resources, and consider their mobile phone to be a useful resource for training during their clinical practice [46,47], rather than a distraction. However, other studies indicate that this may be related to a lack of awareness about their use of mobile phones [12,48]. In terms of self-management of clinical activities, the scores obtained were above average, coinciding with a study conducted among nurses [26].

Data from this study reveal that problematic use of SNSs correlates positively with perceived stress in nursing students. In the same line, the problematic use of SNSs is related to higher perceived stress, consistent with previous studies reporting that SNS overuse generates cognitive overload that elevates stress responses in nursing students [49,50]. Several studies establish a positive relationship between the use of SNSs and distraction in nursing students [10,35]. These data are consistent with those obtained in this study. The findings of this study report that the greater the problematic use of SNSs, the greater the distraction associated with smartphone use during nursing students’ clinical practice. Along these same lines, the problematic use of smartphones has been related to greater distraction during clinical practicums, which can interfere with the quality of care provided to patients [10] and decrease academic performance [51,52]. Furthermore, no significant association was found between perceived stress and smartphone-related distraction, suggesting that distraction during clinical practice may be independent of students’ stress levels [29]. Likewise, problematic use of SNSs is related to lower self-management of nursing students. This association suggests that as SNS use becomes more problematic, students’ capacity to regulate and organize their clinical tasks diminishes [53]. Self-management of clinical activities is essential to ensure optimal performance in the clinical setting [26]. Notably, no significant association was found between smartphone-related distraction and self-management. This finding may be explained by the fact that nursing students in clinical practicums tend to underestimate their own smartphone-induced distraction, even when use is objectively high [8,10]. Consequently, self-management may be regulated by other factors beyond perceived distraction, such as professional accountability or clinical supervision [54]. For these reasons, it is necessary to develop policies that regulate smartphone use in clinical settings in order to reduce the associated risks such as distraction [55,56], loss of relevant clinical information [57], decreased performance [36], and the loss of privacy and patient confidentiality related to problematic use of SNSs [39].

In addition, nursing students indicated that greater self-control decreases perceived stress. This could be due to the fact that self-control influences nurses’ clinical performance, helping them to focus their attention on the task at hand, making them more efficient at work [25], avoiding task accumulation, and thus reducing perceived stress [58]. To the best of our knowledge, no previous study has simultaneously examined SNS use, perceived stress, smartphone distraction, and self-management in nursing students during clinical practice, although there are previous studies linking problematic SNS use to the clinical performance of nurses [19,26]. In this regard, it is necessary to implement educational interventions to improve self-management in nursing students [26], as it is likely that SNS usage habits as students will continue into their professional practice, with the purpose of improving their clinical performance.

The results indicated that the nursing students who carried out their last rotation of practicums in a hospital had greater problematic use of SNSs than students who carried out their last rotation of practicums in a health clinic setting. This could be due to the fact that nursing professionals who work in hospital settings face heavy workloads and argue that they need to use their smartphone to decrease their emotional load and to socialize [48]. Therefore, they connect to SNS in the clinical setting [59]. Regarding gender, men are more likely to have a problematic use of SNSs. This could be due to the fact that WhatsApp is the most used SNS in the study context, being considered in many cases to be the only SNS used by men to interact through groups for personal and professional matters [60].

### 4.1. Limitations

The results of this study should be considered in relation to a number of methodological limitations. Firstly, the study design makes it impossible to establish causal relationships between SNS use, perceived stress, and self-regulation/self-management. Furthermore, the sample was obtained through convenience sampling, and the participants belonged to a single institution, which may limit the representativeness and generalization of the results to other contexts. Likewise, the data were collected through self-report measures, which may lead to social desirability biases. Regarding sex/gender, only the categories female and male were collected, with no additional options (e.g., non-binary/other) or ‘prefer not to say’. On the other hand, the impact of social networks was assessed using a scale focused on the use of WhatsApp, which limits extrapolation to other SNSs; therefore, it would be appropriate to analyze different SNSs and their impact during the clinical practice of nursing students in a differentiated manner.

### 4.2. Future Research Direction

Future longitudinal studies would help establish relationships between the variables analyzed in this study. It would also be a good idea to include objective measures, like assessments by outside observers or tracking time spent on mobile phones and SNSs through mobile apps, to make sure the data in future studies is objective. Finally, it is necessary to further explore the implications for patient safety and quality of care, specifically assessing how distraction and time loss associated with SNS use in the clinical setting may affect student learning and skill acquisition. Similarly, the development of qualitative studies in nursing managers and University lecturers would allow for the identification of specific strategies to regulate and optimize mobile phone use in the clinical and academic settings.

### 4.3. Implications for Clinical Practice

No previous study has explored the impact that the use of SNSs among nursing students in the clinical setting may have on distraction, self-management of clinical activities, and perceived stress. Considering the results of this study, problematic use of SNS may negatively affect their academic and clinical performance. Therefore, it is necessary to develop policies that regulate the use of smartphones in clinical settings in order to reduce the associated risks, such as distraction, loss of relevant clinical information, decreased performance, and loss of patient privacy and confidentiality related to problematic SNS use. Specifically, it is important to develop interventions that do not stigmatize or restrict the use of smartphones in the clinical setting, but rather promote conscious and healthy use. In addition, it is necessary to implement strategies for the prevention of problematic use of new technologies by university institutions and interventions to help nursing students who show problematic use of social media. This could prevent clinical errors, improve the effectiveness of their clinical and academic tasks, optimize their educational process, and ensure patient safety.

## 5. Conclusions

Nursing students have shown a moderate level of problematic use of SNSs. Problematic SNS use was associated with higher perceived stress and distraction and with lower self-management during clinical practicum. Likewise, nursing students who reported a lower level of perceived stress indicated a greater capacity for self-management of clinical activities. The results of the study showed a direct and significant relationship between problematic use of SNSs, spending more time per day using the smartphone, distraction associated with the smartphone during clinical practices, perceived stress, and clinical practice in the hospital setting being higher in male nursing students.

## Figures and Tables

**Table 1:** Sociodemographic characteristics of the sample.

Variable	Mean (*SD*)	Frequency (*f*)	Percentage (%)
Age (years)	23.00 (4.58)		
Sex Male Female		53 287	15.60 84.40
Academic Year 2nd year 3rd year 4th year		106 103 131	31.20 30.30 38.50
Last place practicum was carried out Hospital Health Clinic		271 69	79.70 20.30
Amount of time spent on smartphone per day <1 h/day 1–3 h/day 3–5 h/day >5 h/day		3 14 194 129	0.90 4.10 57.10 37.90

Note: SD, Standard deviation.

**Table 2:** WhatsApp Negative Impact Scale, Perceived Stress Scale, Distraction associated with smartphone use, and Self-Control and Self-Management Scale.

		WANIS	PSS	DSCS	SCMS
WANIS	*r_s_* *p*	-	0.26 0.001	0.25 0.001	−0.19 0.001
PSS	*r_s_* *p*	0.26 0.001	-	0.01 0.88	−0.28 0.001
DSCS	*r_s_* *p*	0.25 0.001	0.01 0.88	-	0.07 0.19
SCMS	*r_s_* *p*	−0.19 0.001	−0.28 0.001	0.07 0.19	-

Note: *r_s_* = Spearman’s correlation coefficient; WANIS, WhatsApp Negative Impact Scale; PSS, Perceived Stress Scale; DSCS, Distraction Smartphone Use during Clinical Practice Scale; SCMS, Self-Control and Self-Management Scale. The significance level was set at *p* < 0.05.

**Table 3:** Factor influencing problematic use of SNSs (WANIS).

Variable	*B*	*SE B*	*β*	*t*	*p*	*CI* 95%
Constant	21.32	5.53		3.86	0.001	[10.45, 32.18]
PSS	0.46	0.13	0.19	3.64	0.001	[0.21, 0.70]
DSCS	1.12	0.27	0.22	4.19	0.001	[0.59, 1.64]
Sex	−5.14	2.31	−0.12	−2.23	0.026	[−9.70, −0.60]
Last place practicum was carried out	−5.66	2.06	−0.14	−2.75	0.006	[−9.70, −1.60]
Daily time spent on smartphone (>5 h/day)	5.79	1.42	0.21	4.06	0.001	[2.98, 8.58]

Note: Results from a multiple linear regression model: *B* = unstandardized coefficient; *SE B* = standard error; *β* = standardized coefficient; *t* = t statistic; *CI* = confidence interval. The significance level was set at *p* < 0.05.

## Data Availability

The raw data supporting the conclusions of this article will be made available by the authors on request. All data supporting the findings of this study are provided within the article. No additional datasets were generated or analyzed during the current study; therefore, there are no supplementary data to share publicly. This study was conducted at the University of Almeria, and due to institutional policies and confidentiality considerations, raw data cannot be made publicly available.

## Public Involvement Statement

No public involvement in any aspect of this research.

## Guidelines and Standards Statement

This manuscript was drafted following the STROBE (The Reporting of Observational Studies in Epidemiology) guidelines for quantitative research [21].
